# Identification of Potential Diagnostic Biomarkers and Biological Pathways in Hypertrophic Cardiomyopathy Based on Bioinformatics Analysis

**DOI:** 10.3390/genes13030530

**Published:** 2022-03-17

**Authors:** Tingyan Yu, Zhaoxu Huang, Zhaoxia Pu

**Affiliations:** Department of Cardiology, The Second Affiliated Hospital, Zhejiang University School of Medicine, Hangzhou 310009, China; yutingyan1993@163.com (T.Y.); huangzhaoxu85@163.com (Z.H.)

**Keywords:** hypertrophic cardiomyopathy, biomarkers, WGCNA, LASSO, GSEA

## Abstract

Hypertrophic cardiomyopathy (HCM) is a genetic heterogeneous disorder and the main cause of sudden cardiac death in adolescents and young adults. This study was aimed at identifying potential diagnostic biomarkers and biological pathways to help to diagnose and treat HCM through bioinformatics analysis. We selected the GSE36961 dataset from the Gene Expression Omnibus (GEO) database and identified 893 differentially expressed genes (DEGs). Subsequently, 12 modules were generated through weighted gene coexpression network analysis (WGCNA), and the turquoise module showed the highest negative correlation with HCM (cor = −0.9, *p*-value = 4 × 10^−52^). With the filtering standard gene significance (GS) < −0.7 and module membership (MM) > 0.9, 19 genes were then selected to establish the least absolute shrinkage and selection operator (LASSO) model, and *LYVE1*, *MAFB*, and *MT1M* were finally identified as key genes. The expression levels of these genes were additionally verified in the GSE130036 dataset. Gene Set Enrichment Analysis (GSEA) showed oxidative phosphorylation, tumor necrosis factor alpha-nuclear factor-κB (TNFα-NFκB), interferon-gamma (IFNγ) response, and inflammatory response were four pathways possibly related to HCM. In conclusion, *LYVE1*, *MAFB*, and *MT1M* were potential biomarkers of HCM, and oxidative stress, immune response as well as inflammatory response were likely to be associated with the pathogenesis of HCM.

## 1. Introduction

Hypertrophic cardiomyopathy (HCM) is a genetic heterogeneous disorder resulting in left ventricular hypertrophy, myocardial hypercontractility, reduced compliance, myofibrillar disarray, and fibrosis [1]. Epidemiologic studies have shown that the prevalence of HCM is estimated to be 0.2% (1 case per 500 persons) in the general population, but much higher (0.5%; 1 case per 200 persons) when both clinical and genetic diagnoses, including those in family members, are taken into account [2]. HCM has been the main cause of sudden cardiac death among adolescents and young adults, especially athletes [3]. Current therapies for HCM have been effective in reducing morbidity; however, it is still hard to reverse this disease. With the advent of embryonic gene editing, it is possible to correct underlying genetic mutations and prevent the transmission of mutations to future generations [1]. Previously, HCM was thought to be mainly associated with genetic variants which occurred in nine sarcomeric genes, especially *MYH7* and *MYBPC3* [3,4]. Nevertheless, there are still a large number of underlying mutations undetermined, and the signaling pathways and regulatory networks underlying the pathogenesis of HCM are also not fully understood [5,6]. Thus, it is vital to explore and identify potential genes and biological pathways related to the pathogenesis of HCM for diagnosis, treatment, and prevention.

Recently, microarray and high-throughput techniques, along with bioinformatics analyses, have promoted the identification of novel key genes and pathways involved in disease pathogenesis. The Gene Expression Omnibus (GEO) database is a public database that contains a large resource of high-throughput gene expression data submitted by researchers around the world. Weighted gene coexpression network analysis (WGCNA) is a systems biology method for describing the correlation patterns among genes across microarray samples, and this method can be employed to identify candidate biomarkers or therapeutic targets [7], which has been widely used in various disease research studies [8,9,10]. The least absolute shrinkage and selection operator (LASSO) is a machine learning method to identify the best feature from high-dimensional data with a high predictive value and low correlation [11], which can significantly improve the accuracy and predictive value of key genes identified from microarray and high-throughput data [12].

**Figure 1:**
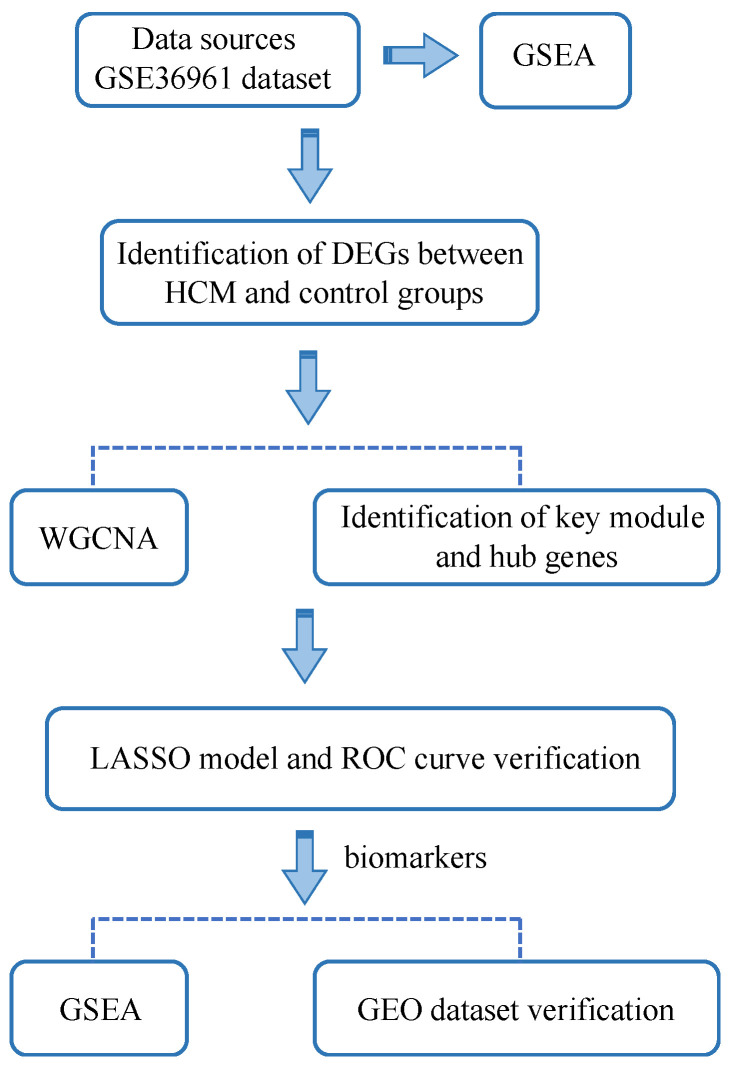
Workflow to identify potential biomarkers and biological pathways in hypertrophic cardiomyopathy (HCM). We firstly obtained differentially expressed genes (DEGs) from GSE36961 dataset, and then conducted WGCNA, LASSO, and GSEA step by step to find potential biomarkers and biological pathways in HCM.

Herein, we obtained the HCM dataset GSE36961 from the GEO database and identified differentially expressed genes (DEGs). Subsequently, WGCNA was conducted to filter key module and hub genes. Furthermore, the above genes were used to establish the LASSO model and three key genes were then identified as biomarkers in HCM. Functional enrichment analysis was also performed to explore the potential biological process in HCM (Figure 1). Such findings could provide insights into the biomarkers and biological pathways in HCM, and may help to diagnose, treat and prevent HCM in the future.

## 2. Materials and Methods

### 2.1. Data Sources and Searches

We searched gene expression profiling datasets for HCM using the term “hypertrophic cardiomyopathy” in the GEO database (https://www.ncbi.nlm.nih.gov/geo/, accessed on 1 December 2021), and the eligible datasets must meet the following inclusion criteria: (i) the dataset should comprise genome-wide expression mRNA microarray data, (ii) the samples were cardiac tissues from HCM and non-HCM controls/healthy subjects, (iii) the organism was restricted to Homo sapiens, and (iv) the sample sizes should be sufficient for analysis. Finally, this study selected GSE36961 based on the GPL15389 platform for further analysis, which included 109 HCM samples and 39 control samples. The utilization of data in the GEO database is free of charge and does not require ethical approval.

### 2.2. Identification of DEGs

The raw data were normalized and used to identify DEGs between HCM and control groups by the “limma” package in R software (version 3.6.0) [7]. Genes with *p*-value < 0.05 and |(log_2_FC)| > 0.5 were considered as DEGs in this study. A volcano plot for DEGs was drawn using “ggplot” package under the R software platform.

### 2.3. WGCNA Network Construction and Hub Genes Identification

The “WGCNA” package in R software was used to construct the gene coexpression network to explore the expression and interaction of DEGs in HCM samples [13]. Firstly, the soft threshold was used to ensure a scale-free network. Subsequently, the network interconnectedness was built by topological overlap matrix (TOM), and gene modules were identified based on the hierarchical clustering method. Next, each first principal component of each gene module was identified as the module eigengenes (MEs). We then looked for correlations between clinical features and MEs [14] and identified the module with the highest correlation with HCM as the key one.

Gene significance (GS) was used to identify the relationships between genes and traits, and module membership (MM) represented the correlation between MEs and gene expression profiles. Genes having high GS and MM in the key module were highly interrelated with the clinical features. In the present study, we selected genes with GS < −0.7 and MM > 0.9 to be hub genes in the key module.

### 2.4. LASSO Model Establishment and Key Genes Identification

By using the “glmnet” package in R software, we constructed the LASSO model based on the gene expression profiles of hub genes. The minimum lambda (lambda.min) and one standard error of the minimum lambda (lambda.1se) were used as references to identify the variables in models, which were two suitable lambda values for modeling. The dataset was randomly divided into train set (60%) and test set (40%) to perform receiver operator characteristic (ROC) curve analysis, which was achieved by utilizing the “ROCR” package in R software, so as to evaluate the stability and sensitivity of the two LASSO models based on lambda.min and lambda.1se in identifying HCM. After that, genes obtained from the LASSO models had their intersection taken to be key genes by Venn diagram [15].

### 2.5. Validation of Key Genes

GSE130036, containing human myocardial tissues from 28 HCM patients and 9 healthy donors based on the GPL20795 platform, was retrieved from the GEO database and severed as a validation dataset. Student’s t-test from the R software was used to compare the key genes expression variances between HCM and control groups, and the “ggplot2” R package was employed to draw plots of the expression of key genes.

### 2.6. Gene Set Enrichment Analysis (GSEA)

GSEA is routinely used to analyze and interpret coordinate pathway-level changes in transcriptomics experiments [16], which was used to investigate the potential mechanisms of the key genes according to their correlations with other coding genes calculated by Spearman’s correlation. We utilized the “clusterProfiler” package in R software to perform GSEA [17], and the visualizations were conducted by using “ggplot2” and “enrichplot” packages in R software. The whole gene expression values of the samples were analyzed based on the h.all.v7.4.entrez.gmt [Hallmarks] gene set database (https://www.gsea-msigdb.org/gsea/msigdb/collections.jsp#H, accessed on 18 December 2021), and gene sets were considered meaningful when they satisfied *p*.adjust-value < 0.05.

## 3. Results

### 3.1. Identification of DEGs between HCM and Control Groups

**Figure 2:**
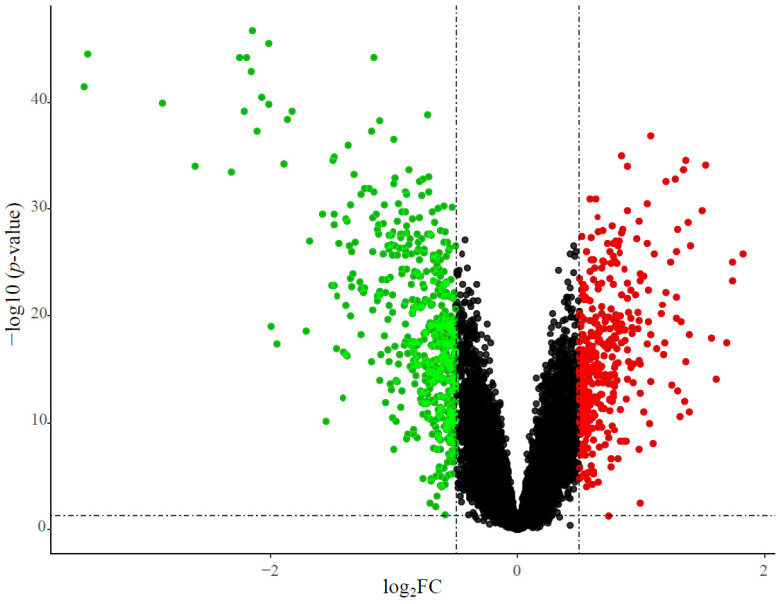
Volcano plots of DEGs. The *x*-axis represents the log2 fold change (log_2_FC) value, and the *y*-axis represents significant difference. The red dots represent significantly upregulated genes, while the green dots represent significantly downregulated genes.

A total of 893 DEGs were identified between patients with HCM and controls, including 387 upregulated genes and 506 downregulated genes. The results were visualized using volcano plots in which each dot corresponded to a gene (Figure 2).

### 3.2. Construction of WGCNA and Identification of Hub Genes

**Figure 3:**
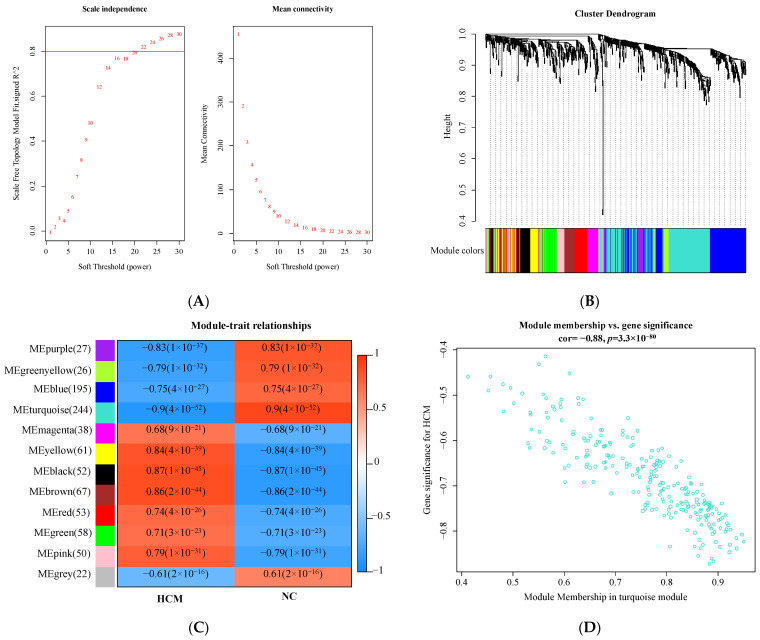
Results of WGCNA. (**A**) Soft threshold analysis. Each power corresponds to scale independence and mean connectivity. A soft threshold of 20 (red horizontal line) was chosen for the construction of the scale-free topology module. (**B**) Cluster dendrogram. Each branch represents one gene, and each color at the bottom represents one coexpression module. (**C**) Module-trait relationships between MEs and clinical phenotype. The upper number in the color grid represents the correlation as well as its *p*-value. Red indicates a positive correlation, and blue indicates a negative correlation. (**D**) The scatterplot of module membership and gene significance in turquoise module.

WGCNA was conducted on the coexpression network of the 893 DEGs. In the present study, 20 was selected to be the soft threshold for the construction of the scale-free topology module (Figure 3A). A gene coexpression network was then constructed based on the hierarchical clustering method, and 12 modules were generated (Figure 3B). Moreover, we also performed a correlation analysis between the modules and phenotypes of clinical traits. The turquoise module had the highest negative correlation with HCM (cor = −0.9, *p*-value = 4 × 10^−52^), while the black module exhibited the highest positive correlation with HCM (cor = 0.87, *p*-value = 1 × 10^−45^) (Figure 3C). We then focused on the turquoise module for further study, which had the greatest absolute correlation among all modules. Figure 3D displays the correlation analysis between MM and GS in the turquoise module, whose correlation value was −0.88 (*p*-value = 3.3 × 10^−80^). If the genes in the turquoise module met the requirements of GS < −0.7 and MM > 0.9, they would be designated as hub genes and used for further analysis. Finally, 19 genes satisfied the above requirements, which were *CD163*, *FCER1G*, *S100A9*, *FPR1*, *MAFB*, *LYVE1*, *SERPINA3*, *CTSC*, *ALOX5*, *CMTM7*, *LCP1*, *SLA*, *WAS*, *HAVCR2*, *CORO1A*, *HCK*, *MT1M*, *HCLS1*, and *EBI2*, respectively.

### 3.3. Establishment of LASSO Model and Identification of Key Genes

**Figure 4:**
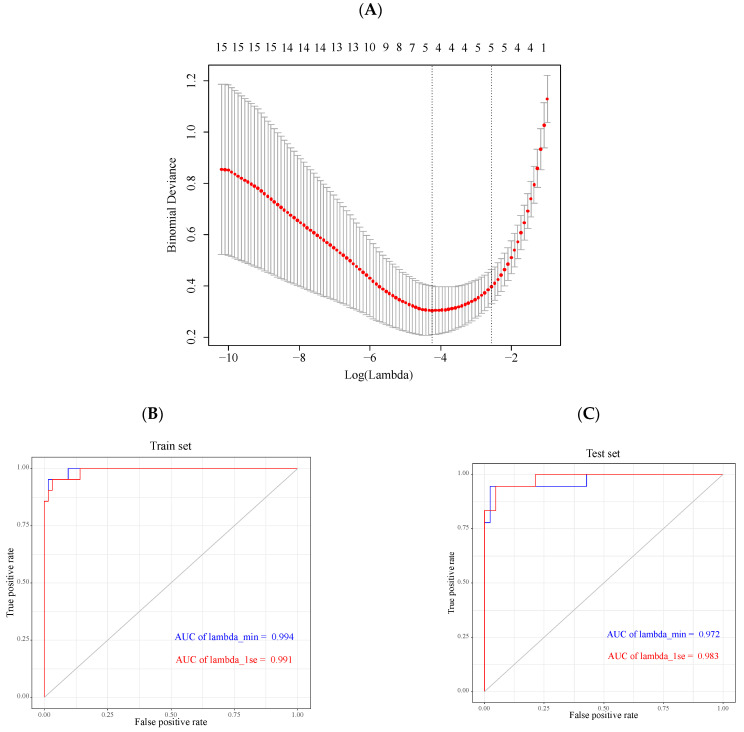

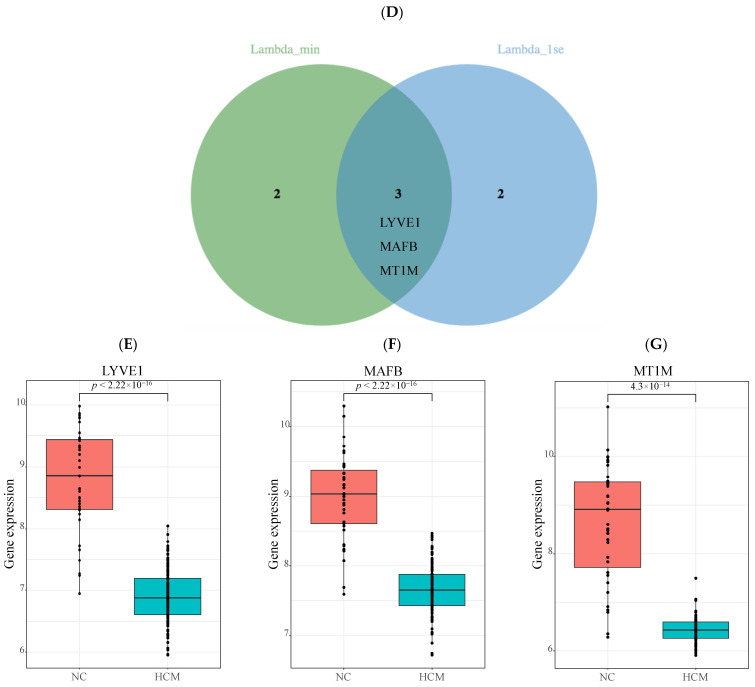
LASSO model establishment and key genes identification. (**A**) The optimal lambda selection in LASSO model. The left dotted line represented lambda.min, and the right dotted line represented lambda.1se. (**B**) Receiver operator characteristic (ROC) curves in train set. The blue line represented the ROC curve calculated based on the value of lambda.min, and the red line represented the ROC curve calculated based on the value of lambda.1se. (**C**) ROC curves in test set. (**D**) Three common key genes screened from two LASSO models are shown in Venn diagram. (**E**–**G**) Differential expressions of key genes in GSE36961 dataset. (**E**) The differential expression of *LYVE1* between HCM and control groups; (**F**) The differential expression of *MAFB* between HCM and control groups; (**G**) The differential expression of *MT1M* between HCM and control groups. All *p* < 0.001.

The 19 genes selected from the turquoise module were used to establish the LASSO models based on the values of lambda.min = 0.01431306 and lambda.1se = 0.07638451 (Figure 4A). The area under the curves (AUCs) according to lambda.min and lambda.1se in the train set were 0.994 and 0.991 (Figure 4B), while the AUCs in the test set became 0.972 and 0.983, respectively (Figure 4C). In the LASSO model based on lambda.min, five genes were then screened, including *CORO1A*, *LYVE1*, *MAFB*, *MT1M* and *WAS*, and another five genes were also filtrated from the LASSO model based on lambda.1se, containing *LYVE1*, *MAFB*, *MT1M*, *S100A9*, and *SERPINA3*. A Venn diagram was then developed to take the intersection of the above genes to identify key genes in HCM, which were *LYVE1*, *MAFB*, and *MT1M*, respectively (Figure 4D). These genes were downregulated in HCM patients compared to controls (all *p* < 0.001) (Figure 4E).

### 3.4. Validation of Key Genes

**Figure 5:**
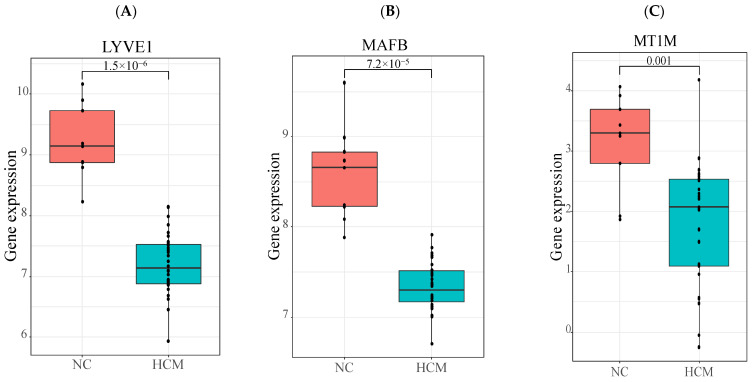
Validation of the differential expressions of three key genes in GSE130036 dataset. (**A**) The differential expression of *LYVE1* between HCM and control groups; (**B**) The differential expression of *MAFB* between HCM and control groups; (**C**) The differential expression of *MT1M* between HCM and control group. All *p* < 0.01.

The GSE130036 dataset was adopted for data validation. The distribution of expression levels of *LYVE1*, *MAFB*, and *MT1M* was shown in Figure 5, and their expressions were significantly decreased in HCM patients compared to those in control groups (all *p* < 0.01), consistent with the situations observed in the GSE36961 dataset.

### 3.5. Signaling Pathways Identified by GSEA

**Figure 6:**
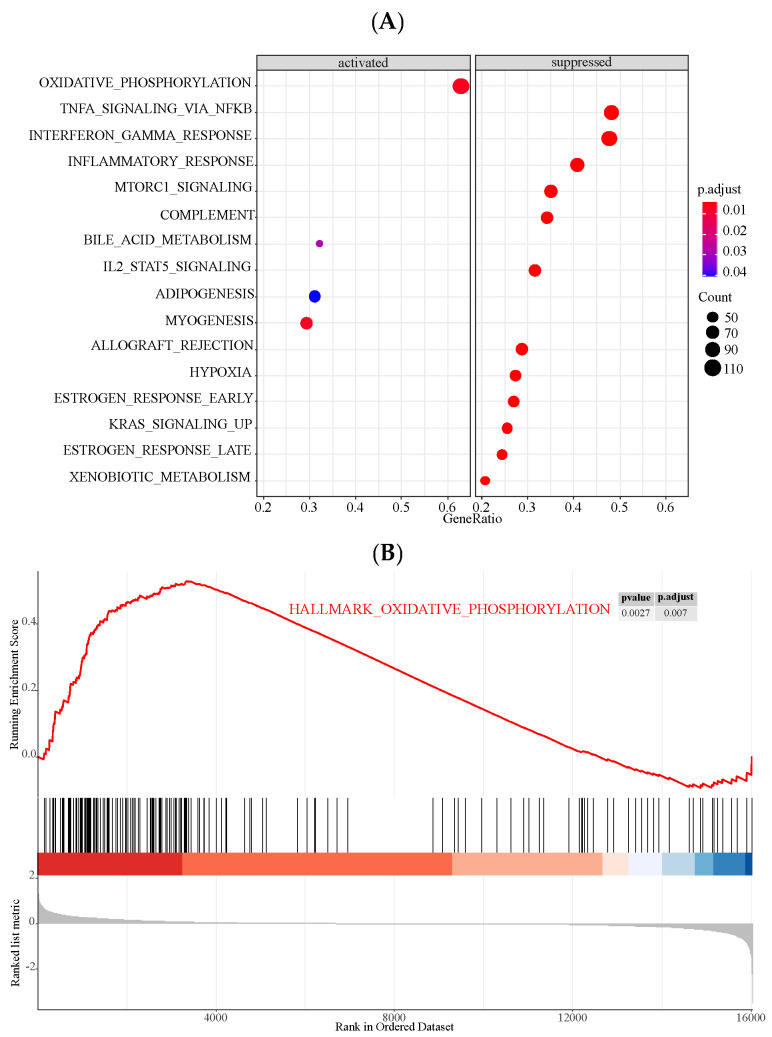

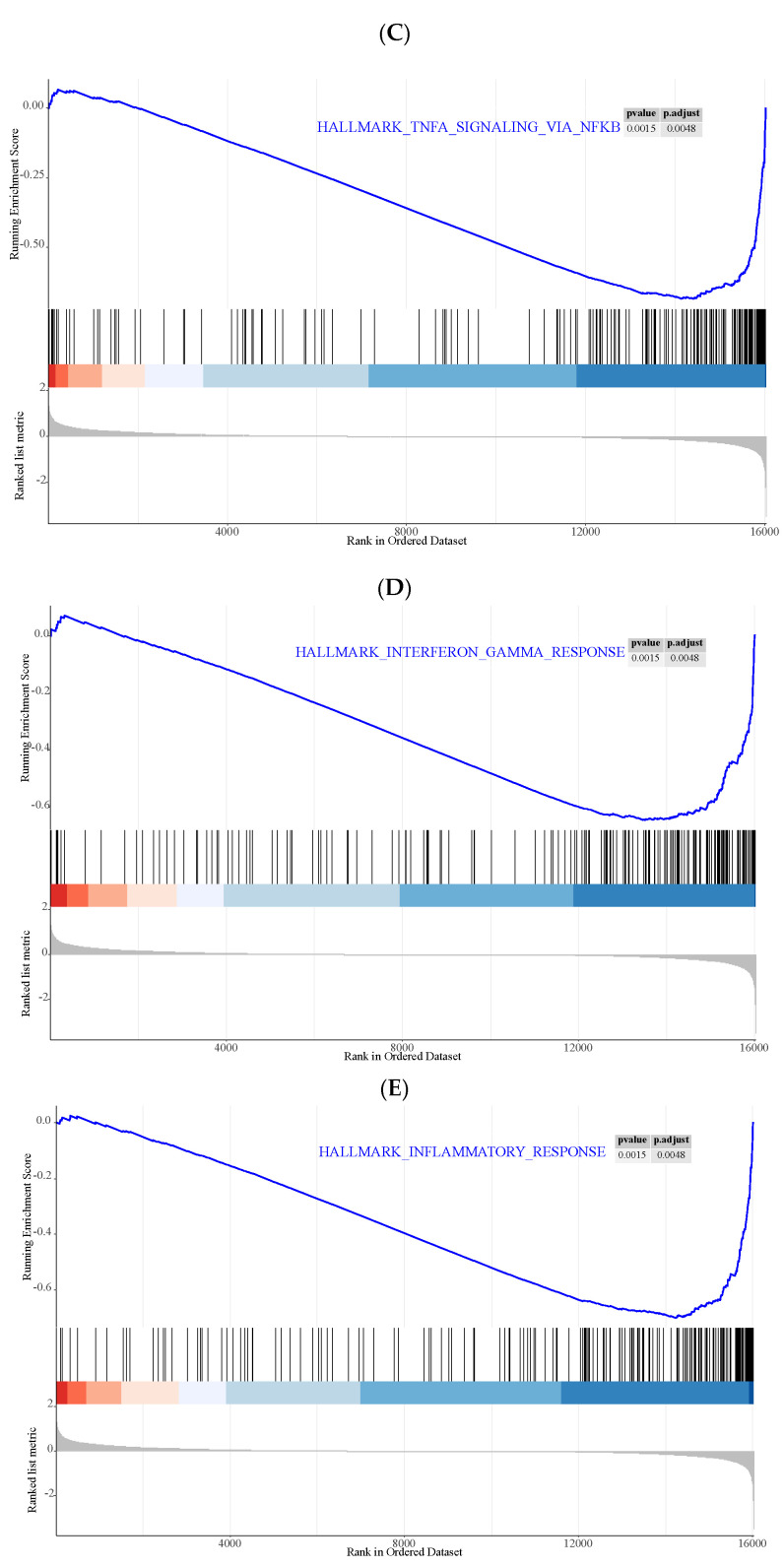
Results of GSEA in HCM of GSE36961 database. (**A**) Enrichment plots of HCM. The color intensity of the nodes represents the enrichment degree of pathways. The horizontal axis is gene ratio representing the proportion of differential genes in the whole gene set. The dot size represents the gene counts in a certain pathway. Oxidative phosphorylation pathway ranked first among the activated pathways, while tumor necrosis factor alpha-nuclear factor-κB (TNFα-NFκB), interferon-gamma (IFNγ) response, and inflammatory response ranked the top three among the suppressed pathways. (**B**) Enrichment plot of genes involved in oxidative phosphorylation. (**C**) Enrichment plot of genes involved in TNFα-NFκB. (**D**) Enrichment plot of genes involved in IFNγ response. (**E**) Enrichment plot of genes involved in inflammatory response.

GSEA was firstly conducted using data from HCM patients and controls in the GSE36961 database, and the results are presented in Figure 6. We found that the oxidative phosphorylation pathway was mainly enriched in the HCM group among the activated pathways, while tumor necrosis factor alpha-nuclear factor-κB (TNFα-NFκB), interferon-gamma (IFNγ) response, and inflammatory response were three suppressed pathways predominately enriched in HCM.

**Figure 7:**
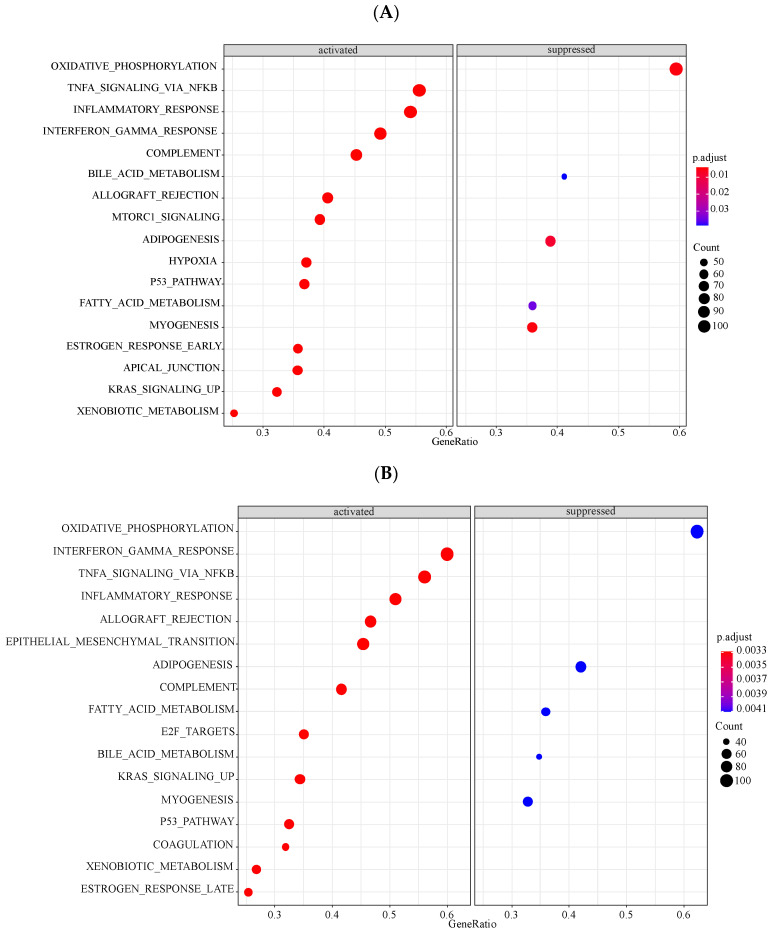

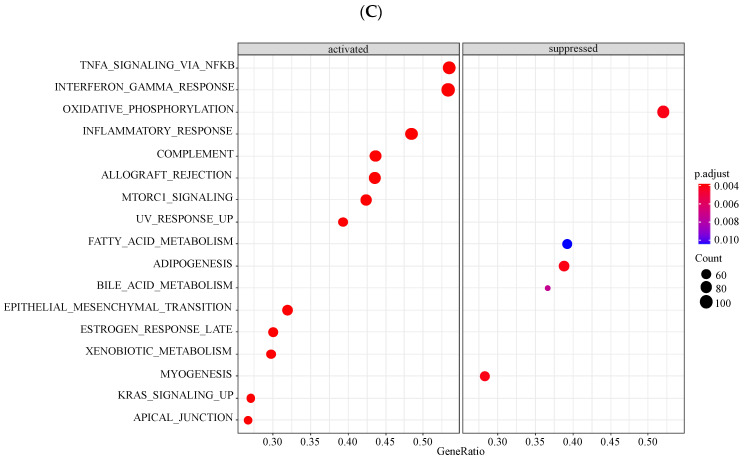
GSEA results of three key genes. (**A**) Enrichment plots of *LYVE1*. (**B**) Enrichment plots of *MAFB*. (**C**) Enrichment plots of *MT1M*. Oxidative phosphorylation pathway ranked first among the suppressed pathways no matter in *LYVE1*, *MAFB*, or *MT1M*, whereas TNFα-NFκB, IFNγ response, and inflammatory response pathways ranked the top three among the activated pathways.

Next, we further explored the potential signaling pathways related to *LYVE1*, *MAFB*, and *MT1M* based on their positively or negatively coexpressed genes (Tables S1–S3). The results showed that the oxidative phosphorylation pathway was the predominant suppressed pathway no matter in *LYVE1*, *MAFB*, or *MT1M*, whereas TNFα-NFκB, IFNγ response, and inflammatory response pathways were mostly enriched in three key genes among the activated pathways (Figure 7 and Figures S1–S3).

## 4. Discussion

In the present study, a comprehensive analysis of potential biomarkers and biological processes was carried out using gene profiles from patients with HCM and healthy controls. We have detected 387 upregulated genes and 506 downregulated genes in the GSE36961 dataset, and subsequently generated 12 modules in WGCNA. The turquoise module was the one that had the highest correlation with HCM, and then it was chosen for further analysis. According to results from the LASSO models, *LYVE1*, *MAFB*, and *MT1M* were finally identified as potential biomarkers in HCM. The three key genes were all downregulated in HCM, as well as in the validation dataset GSE130036. Additionally, we explored the latent biological mechanism by GSEA, showing oxidative phosphorylation, TNFα-NFκB, IFNγ response, and inflammatory response were four pathways possibly participating in the pathogenesis of HCM.

GSE36961 is a popular dataset in bioinformatics analysis due to its abundant samples, containing more than 100 HCM patients. Previous research was mostly inclined to use the GSE36961 dataset as a validation dataset or to construct lncRNA-miRNA-mRNA networks with other lncRNA and miRNA datasets [18,19,20,21,22,23]. In addition, the GSE36961 dataset was also used to combine with other mRNA datasets to obtain DEGs for further study [24,25]. A few studies mainly focused on the GSE36961 dataset to analyze the mechanism involved in HCM. Hu et al. have explored GSE36961 by using Gene Ontology (GO) analysis and protein–protein interaction (PPI) network [26]. In our study, we chose WGCNA, LASSO, and GSEA to identify the potential biomarkers and biological pathways in HCM and obtained different results ultimately, which provided a new way of data mining for the GSE36961 dataset.

As one of the key genes found in this study, *LYVE1* (lymphatic vessel endothelial hyaluronan receptor-1) is a homolog of the CD44 hyaluronan receptor and a member of the Link protein family, with high expression in the overlapping junctions of lymphatic capillaries [27]. Consistent with another bioinformatics analysis based on the GSE36961 and GSE141910 datasets [24], we both observed decreased expression of *LYVE1* in HCM patients. Previous research showed that deletion of *Lyve1* in mice would prevent docking and transit of leukocytes through the lymphatic endothelium, resulting in exacerbation of chronic inflammation and long-term deterioration of cardiac function [28]. Additionally, another study based on single-cell mRNA sequencing technology demonstrated that the acute depletion of *LYVE1^high^* cardiac resident tissue macrophages (RTMs) using a mouse model of inducible macrophage depletion during the induction of fibrosis could exacerbate vessel permeability, immune cell infiltration, and collagen deposition, implying *LYVE1* might participate in restraining inflammation and fibrosis [29]. In addition, Jiang et al. pointed out that the elevated lymphatic microvessel density (LMVD) stained immunohistochemically with LYVE1 antibodies had a close relationship with ventricular septal fibrosis in hypertrophic obstructive cardiomyopathy (HOCM) patients [30]. Above all, since we observed a decreased expression of *LYVE1* in HCM patients, the existing research hinted at the possibility that the downregulation of *LYVE1* affected the inflammatory process and impaired its anti-fibrotic function, thereby contributing to the development and progression of HCM. However, we still need further study to confirm such a hypothesis.

*MAFB* (MAF basic region leucine zipper transcription factor B) is another key gene discovered in our research and belongs to the large Maf family. *MAFB* is specially expressed in glomerular podocytes, macrophages, and osteoclasts and acts as a switch to initiate gene transcription, participating in physiological processes such as cell proliferation, differentiation, apoptosis, and migration [31]. Tani-Matsuhana S et al. demonstrated that *MAFB* knockdown would reduce the cardiac neural crest stream, whose migration to the heart was critical for the formation of cardiac outflow tract and ventricles [32]. Additionally, it has been found that when induced by external stimulus, *MAFB* could be regulated by inflammatory signals such as microRNAs or cytokines and might play a role in anti-inflammation in various macrophage-related diseases [33]. The expression level of *MAFB* was downregulated in HCM patients in our study; however, relevant published research regarding the relationship between *MAFB* and HCM has not been found up to now. The present study is the first one focusing on the role of *MAFB* in HCM, and we wonder whether *MAFB* affects HCM by influencing the congenital development of the myocardium or by involving the inflammatory process induced by an environmental stimulus; regardless, further study is definitely required to investigate the concrete mechanism.

MT1M (Metallothionein 1M) is an isoform of Metallothioneins (MTs), which are a family of low-molecular-weight (ranging from 6 to 7 kDa) and cysteine-rich proteins that bind to heavy metals through the cysteine thiol group [34]. Particularly, MT1M participates in zinc homeostasis via tightly controlling zinc concentrations by zinc-thiol binding [35]. It has been reported that MTs would protect cells against oxidative stress by scavenging free radicals and releasing zinc into the cytoplasm [36,37]. Thus, the reduced availability of MT1M would be expected to enhance oxidative stress by altered regulation of superoxide radicals, as well as by effects on zinc metabolism [37]. Additionally, previous research also showed that the expression of *MT1M* was regulated by pro-inflammatory cytokines, indicating *MT1M* might participate in the inflammation process as well [38]. The relationship between *MT1M* and HCM has not been reported before; therefore, in-depth research is needed to reveal the underlying mechanism of *MT1M* on HCM.

In addition, the results emerging from GSEA demonstrated that the oxidative phosphorylation pathway was enriched in the suppressed pathways in *LVYE1*, *MAFB*, and *MT1M*, whereas TNFα NFκB, IFNγ response, and inflammatory response pathways were enriched in the activated pathways. Notably, the expressions of the above three genes were downregulated in HCM, and these four pathways showed an opposite tendency in HCM, with oxidative phosphorylation pathway activated, TNFα-NFκB, IFNγ response, and inflammatory response pathways inhibited.

Oxidative phosphorylation is an important process participating in the generation of reactive oxygen species (ROS) induced by electron transfer in mitochondria [39], and it has been reported that enhanced mitochondrial oxidative stress due to increased ROS level was possibly related to the occurrence of feline HCM [40]. As mentioned above, MTs would protect cells against oxidative stress and the reduced availability of MT1M could oppositely enhance oxidative stress [37]. In addition, other research on diabetic nephropathy found that overexpression of *MAFB* in diabetic mice would decrease the level of the oxidative stress marker 8-hydroxydeoxyguanosine in urine [41]. Therefore, we speculated whether the inhibition of oxidative phosphorylation by *M1TM* and *MAFB* could be reversed due to their downregulated expressions in HCM, then inducing the occurrence of HCM by affecting the downstream oxidative stress, which needs validation in further study.

**Figure 8:**
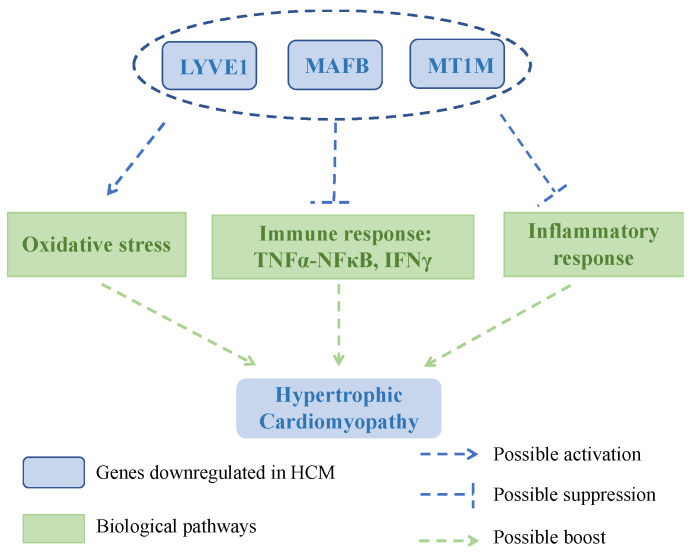
A scheme illustrating the potential relationships between *LYVE1*, *MAFB*, *MT1M* and the possible biological pathways in HCM. The downregulations of *LYVE1*, *MAFB*, and *MT1M* might induce the development and progression of HCM by activating oxidative stress while suppressing immune response and inflammatory response.

TNFα and IFNγ were originally found to be produced by inflammatory cells and to play important roles in the immune system [42]. NFκB is a well-known downstream of TNFα [43], and it has proved active in heart’s lymphatic system confirmed by LYVE1 immunohistochemistry [44]. Another study on human blood CDc1^+^ dendritic cells showed that the CD5^low^ subset expressed a high level of MAFB, as well as produced a high level of IFNγ [45]. Together, it indicated that *LYVE1* and *MAFB* might participate in immune response in the pathogenesis of HCM. Notably, Zhang et al. have proposed that LYVE1^+^ macrophages might act as a bridge between the heart’s immune system and lymphatic system by using immune infiltration analysis [24], whose conclusion was similar to our findings that two immune response-related pathways, TNFα-NFκB and IFNγ response, were enriched in the GSEA analysis of *LYVE1*, but with different bioinformatics methodologies. Apart from immune-response-related pathways, we also detected inflammatory response pathways in this study, which represented a chain of organized, dynamic reactions with specific humoral secretions, consisting of proinflammatory systems and anti-inflammatory systems [46]. As discussed above, *LYVE1*, *MAFB*, and *MT1M* all possibly participated in the inflammatory process; particularly, *LYVE1* and *MAFB* might play roles in the anti-inflammatory process, while we showed the inflammatory process pathway was activated in these three genes. Previous research showed that proinflammatory and anti-inflammatory cytokines were both raised in HCM patients, and even the same cytokine performed opposite levels in different observations [47], indicating it was a complex inflammatory process in the pathogenesis of HCM. The GSEA results reminded us that the pathogenesis of HCM was likely to relate to oxidative stress, immune response, and inflammatory response. A scheme is shown in Figure 8 to illustrate the potential relationships between *LYVE1*, *MAFB*, *MT1M*, and the possible biological pathways, providing orientation for further research.

However, there were some limitations in our study. Firstly, it should be noted that this research was based on bioinformatics analysis from the transcriptomic profiles of the public database, which may be different from actual scenarios. Secondly, the genes and biological pathways screened in this study were not further investigated. Hence, experimental studies to elucidate the concrete mechanisms in HCM are necessary in the future.

## 5. Conclusions

In summary, this study identified *LYVE1*, *MAFB*, and *MT1M* as biomarkers in the development and progression of HCM using the WGCNA method and the LASSO model, and pointed out that the pathogenesis of HCM was likely to relate to oxidative stress, immune response, and inflammatory response by functional enrichment analysis. The biomarkers and pathways identified in this study may provide valuable information for further study of the mechanism of HCM, and are promising to be applied in precision medicine to improve the diagnosis, treatment, and prevention of HCM in the future.

## Data Availability

The data used in this study are from the GSE36961 and GSE130036 datasets of the GEO database.

## Supplementary Materials

The following supporting information can be downloaded at: https://www.mdpi.com/article/10.3390/genes13030530/s1, Figure S1: GSEA results of *LYVE1*; Figure S2: GSEA results of *MAFB*; Figure S3: GSEA results of *MT1M*; Table S1: Genes correlated with *LYVE1* in HCM; Table S2: Genes correlated with *MAFB* in HCM; Table S3: Genes correlated with *MT1M* in HCM.
