# Supplementary material for: Identification of Potential Diagnostic Biomarkers and Biological Pathways in Hypertrophic Cardiomyopathy Based on Bioinformatics Analysis
Source: Genes (Basel). 2022 Mar 17;13(3):530. doi: 10.3390/genes13030530 (PMC8951232; doi:10.3390/genes13030530)
Supplement: Supplementary file 1 [file genes-13-00530-s001.zip › genes-1608505-supplementary/Supplementary files-proof/Figure S3. GSEA results of MT1M.pdf]

(A)

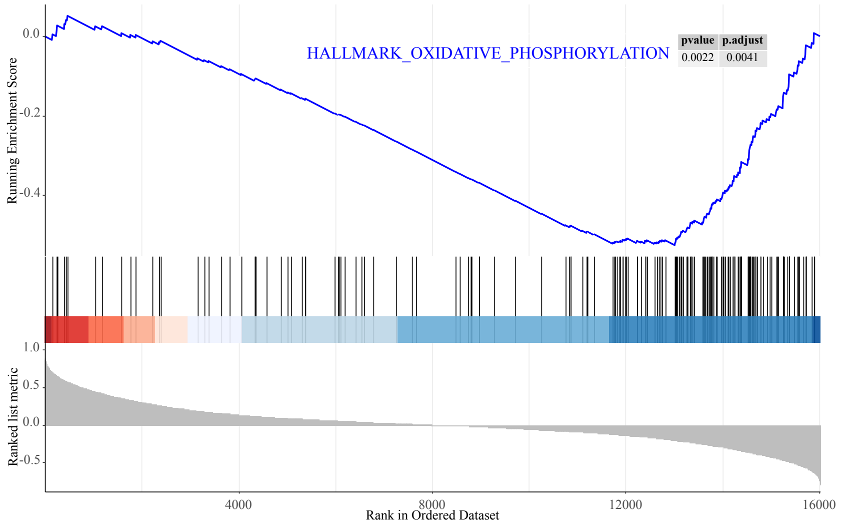

HALLMARK_OXIDATIVE_PHOSPHORYLATION
pvalue p.adjust
0.0022 0.0041
Running Enrichment Score
0.0
-0.2
-0.4
Ranked list metric
1.0
0.5
0.0
-0.5
4000
8000
12000
16000
Rank in Ordered Dataset

(B)

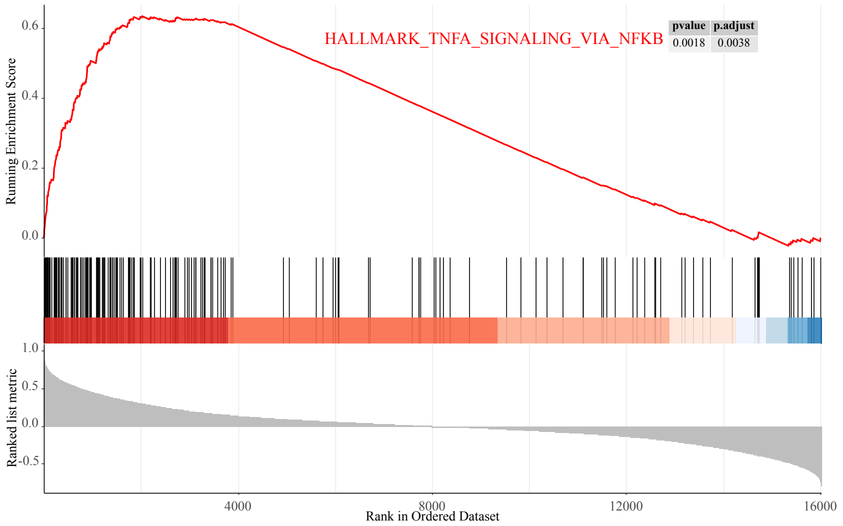

HALLMARK_TNFA_SIGNALING_VIA_NFKB
pvalue p.adjust
0.0018 0.0038
Running Enrichment Score
0.6
0.4
0.2
0.0
Ranked list metric
1.0
0.5
0.0
-0.5
4000
8000
12000
16000
Rank in Ordered Dataset

(C)

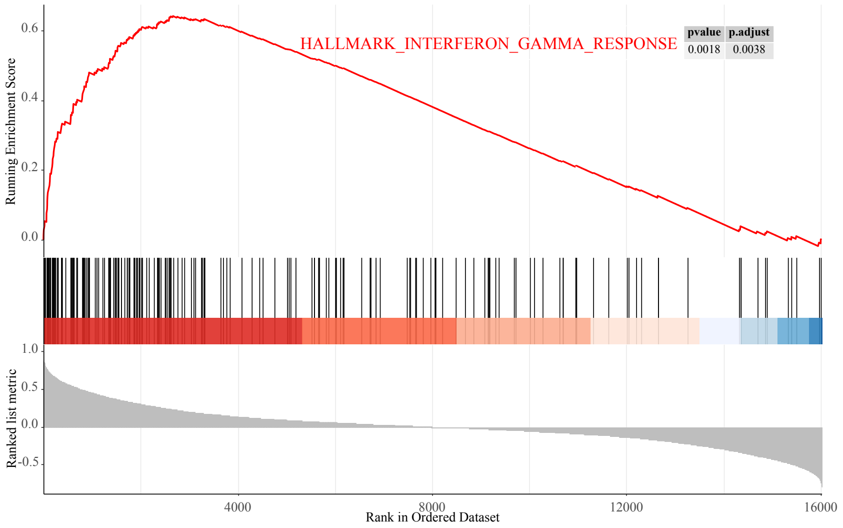

HALLMARK_INTERFERON_GAMMA_RESPONSE
pvalue p.adjust
0.0018 0.0038
Running Enrichment Score
0.6
0.4
0.2
0.0
Ranked list metric
1.0
0.5
0.0
-0.5
4000
8000
12000
16000
Rank in Ordered Dataset

(D)

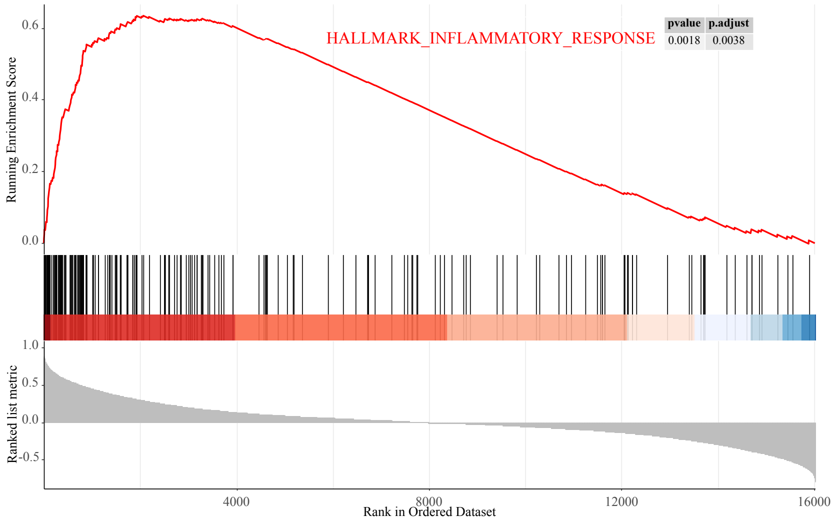

Figure S3. GSEA results of MT1M. (**A**) Enrichment plot of genes involved in oxidative phosphorylation. (**B**) Enrichment plot of genes involved in TNFα-NFκB. (**C**) Enrichment plot of genes involved in IFNγ response. (**D**) Enrichment plot of genes involved in inflammatory response.
